# Synchronous testicular liposarcoma and prostate adenocarcinoma: a case report

**DOI:** 10.1186/1757-1626-3-27

**Published:** 2010-01-14

**Authors:** Umut Demirci, Suleyman Buyukberber, Asli Cakir, Banu Ozturk, Nalan Akyurek, Basak Unver, Meltem Baykara, Mustafa Benekli, Ugur Coskun

**Affiliations:** 1 Department of Medical Oncology Gazi University Medical Faculty, 06500, Beşevler, Ankara, Turkey; 2 Department of Pathology Gazi University Medical Faculty, 06500, Beşevler, Ankara, Turkey

## Abstract

Prostate adenocarcinoma is the most common malignancy and the second leading cause of cancer related deaths in men. Testicular liposarcomas are uncommon soft tissue neoplasms. We report coexistence of prostate cancer and testicular liposarcoma in a 69 year-old-man because while orchiectomy endications are decreasing day by day, these second malignancies should not be missed.

## Introduction

Prostate adenocarcinoma is the most common malignancy in men and also the leading cause of cancer related death. Sarcomas of the genitourinary tract account for 5% of these cases and 2% of all urological tumors. The testis is the most commonly involved urological site and accounts for approximately 30% of all genitourinary sarcomas [1].

Testicular liposarcomas are uncommon soft tissue neoplasms. Liposarcomas, account for 20-50% of sarcomas, seen in the paratesticular region [2,3]. Preoperative diagnosis is infrequent, these tumours sometimes present in elderly adults with a palpable mass of the inguinal canal or scrotum. These tumours may arise from the spermatic cord, fatty tissue or malignant transformation of a pre-existing lipoma [4]. Three pathologic categories of liposarcoma are recognized; well differentiated, myxoid/round cell and pleomorphic. Most paratesticular liposarcomas are well differentiated [5,6]. They frequently have a good prognosis because of their low grade of malignancy [7].

The relative rarity of adult testicular sarcomas has meant that the information used to guide treatment is based on previous experiences from various groups of few patients. Treatment options have invariably been evaluated in previous studies [8,9]. It has a high rate of survival over 5 years [10].

## Case presentation

**Figure 1:**
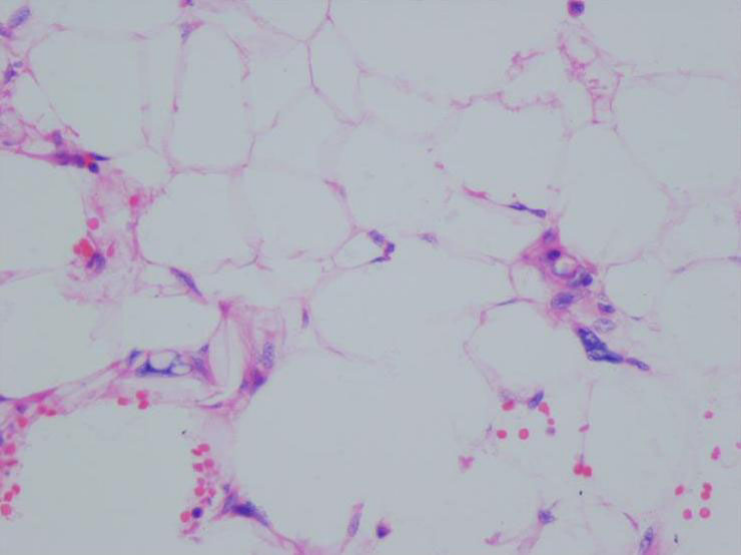
**Acinar type prostate adenocarcinoma, Gleason score 2+3**.

**Figure 2:**
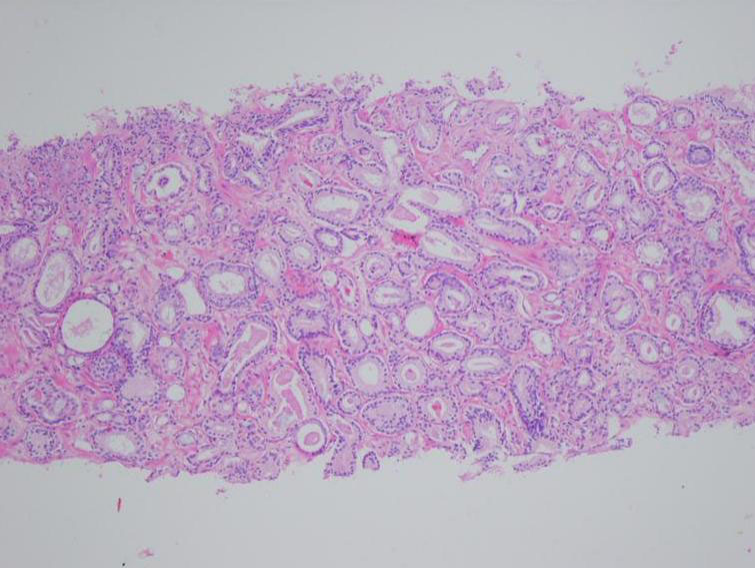
**Atypic lypoblasts, propelled to edge nucleusus with vesicular and notched cytoplasm (HE ×40)**.

In 2005; during a 69-year-old Asian origin Turkish man's routine controls, elevated prostate spesific antigen (PSA) levels was determined. His prostate biopsy appointed as adenocarcinoma with a score of Gleason 2+3 (Figure 1). He has type II diabetes mellitus and hypertension. There was no attribute in his family story and systemic investigation. An abdominal CT scan evaluated prostate's dimensions as 47 × 37 × 33 mm and no additional pathological evidance was determined. In bone scan imaging, increased uptake of right tenth rib and left seventh rib were interpreted as malignancy. It was accepted as stage IV prostate carcinoma and radical orchiectomy was applied. In its microscopic investigation where left testis was normal, low grade liposarcoma was appointed in right testis (Figure 2).

In computed tomography scan of chest, no positive finding was determined. Radiation theraphy with 70 Gy was employed to prostate between July-September 2006. Because of obtaining synchroneous liposarcoma, adjuvant systemic therapy was given as four cure of IMA (Adriamycin (60 mg/m^2^) 1^st^, İfosfamide (2.5 g/m^2^) 1^st^, 2^nd^, 3^rd^, Mesna (2.5 g/m^2^) 1^st^, 2^nd^, 3^rd ^days of every 21 day) in October-December 2006. In September 2008, his PSA and fPSA levels were <0.002 and no differentiation was determined in bone scan imaging.

## Discussion

In recent years, hormone therapy (ADT) has been more choosen than surgical treatment for hormonal deprivation. Like our case; the second and important malignencies could be missed. While orchiectomy endications are decreasing day by day, these second malignancies should not be missed. Our purposes in this case are to show the coexistence of prostate cancer and rare testicular neoplasm ‘liposarcoma’ and evaluate the treatment with literature.

The current guidelines recommends castration alone with either an orchiectomy or LHRH agonists. Patients with widely advanced and metastatic prostate cancer were previously treated with bilateral orchiectomy. Since the introduction of LHRH agonist therapy, surgical intervention has been practiced less often [11]. In our case if medical hormonal therapy was chosen, the diagnose of liposarcoma would be determined later.

A recent meta-analysis included 22 trials with 5710 advanced prostate cancer patients, applied both medical castration and bilateral orchiectomy or one of them alone. Statistically no significant survival advantage was found with combined androgen blockage [12]. Although ADT is associated with high responses, its curative potential is limited and unfortunately nearly all patients develop resistance to hormonal therapy. In a study, 938 locally advanced or asymptomatic metastatic prostate cancer patients received treatment with orchiectomy or LHRH agonist either immediately or after symptoms occurred. Development of disease releated event was twice as common in the deferred-treatment group [13].

Testicular liposarcomas are uncommon cases in the literature. Its coexistence with another malignancy is rare. Although prostate cancer patients have higher risk of a second cancer than general population. The most frequently observed second malignancies in prostate cancer patients include carcinomas of the bladder, stomach, colon and lesser cutaneous and hematolymphoid malignancies The cause of death was always from the second neoplasia [14-16]. In 312 prostate cancer patients, 60 multiple malignant neoplasms (MMNs) was diagnosed. In 13 of them, prostate cancer and other malignancies were diagnosed simultaneously [17]. Another study with 392 patients who were treated for urologic cancers, 42 cases diagnosed with MMNs and only 16 patients (35%) had synchronous MMNs. The incidence of prostatic cancer was higher than other single MMNs associated with genitourinary organs. In this study, the incidence of MMNs with genitourinary cancer was as high as 10.6% and the prognosis of these patients was poor [18]. In 397 patients with urinary system tumors, second malignancy were determined in 29 patients (6.6%) and 21 of them had urological malignancy with the incidence of 6.1%. Another current study follows up with 983 patients who were diagnosed as prostate cancer and whom 106 (11.5%) developed a MMN for sixteen years. Uriary bladder cancer and malignant lymphoma occured in these patients [19].

In our case because liposarcoma was determined after radical orchiectomy, we decided the treatment choice as radiotherapy and four cycles of IMA as a chemotherapy regiment. The optimum local and systemic treatment for these tumours remains controversial, but there is a general consensus that all testicular sarcomas in adults should be managed with radical orchiectomy. Adjuvant locoregional radiation and/or chemotherapy apparently diminishes the risk of local relaps [8,9,20].

## Consent

Written informed consent was obtained from the patient for publication of this case report and any images. A copy of the written consent is available for review by the editor-in-Chief of this journal.

## Competing interests

The authors declare that they have no competing interests.

## Authors' contributions

UD conceived the study. AC, BO, NA, BU, MB and UC performed the literature review. UD, and SB edit and coordinated the manuscript. All authors read and approved the final manuscript.
